# UNICEF Report: enormous progress in child survival but greater focus on newborns urgently needed

**DOI:** 10.1186/1742-4755-11-82

**Published:** 2014-12-06

**Authors:** Tessa Wardlaw, Danzhen You, Lucia Hug, Agbessi Amouzou, Holly Newby

**Affiliations:** Data and Analytics Section, Division of Data, Research, and Policy, United Nations Children’s Fund (UNICEF), 3 United Nations Plaza, New York, NY10017 USA

**Keywords:** Child mortality, Under-five mortality, Neonatal mortality, Newborns, Quality of care, A Promise Renewed

## Abstract

The world has made enormous progress in improving child survival since 1990, reducing the under-five mortality rate by nearly half from 90 to 46 deaths per 1,000 live births in 2013. Currently, the global under-five mortality rate is falling faster than at any other time over the past two decades. Yet, progress is insufficient to meet the Millennium Development Goal 4 (MDG 4) which calls for reducing the under-five mortality rate by two-thirds between 1990 and 2015. If current trends continue in all countries, the world will not meet the target until 2026, 11 years behind schedule. To accelerate progress in child survival, focusing on the newborn is critical since the share of all under-five deaths occurring in the neonatal period (the first 28 days of life) is increasing. Globally, 44 per cent of the 6.3 million under-five deaths occurred in the neonatal period in 2013. Many of these deaths are easily preventable with simple, cost-effective interventions administered before, during and immediately after birth. However, UNICEF’s analysis reveals a remarkably high degree of variability in the utilization and quality of services provided to pregnant women and their babies. Furthermore, quality care is grossly lacking even for babies and mothers in contact with the health system. The latest levels and trends in child mortality as well as the coverage and quality of key maternal and newborn care from pregnancy through childbirth and the postnatal period are the subject of the new UNICEF report *Committing to Child Survival*: *A Promise Renewed Progress Report 2014* released recently in September.

**Electronic supplementary material:**

The online version of this article (doi:10.1186/1742-4755-11-82) contains supplementary material, which is available to authorized users.

A recent UNICEF report *Committing to Child Survival: A Promise Renewed Progress Report 2014*[1] reiterates the significant progress made in improving child survival, using data generated by the UN Inter-agency Group for Child Mortality Estimation [2]. The global under-five mortality rate has declined by almost half, dropping from 90 deaths per 1,000 live births in 1990 to 46 per 1,000 in 2013 (Figure 1). The world is reducing under-five mortality faster than at any other time during the past two decades, with the global annual rate of reduction more than tripling since the early 1990s. The report also highlights that the global advance in child survival continues to elude many of the world’s youngest and most vulnerable children and much still remains to be done to end preventable child deaths. In 2013, of the 6.3 million children who died before their fifth birthday, 16 per cent took their first and final breath on the day they were born. Altogether, 44 per cent died during the first 28 days of life – the neonatal period. The report stresses the critical importance of accelerating progress in saving the lives of newborns with simple, cost-effective interventions as well as quality care before, during and immediately after birth.

**Figure 1:**
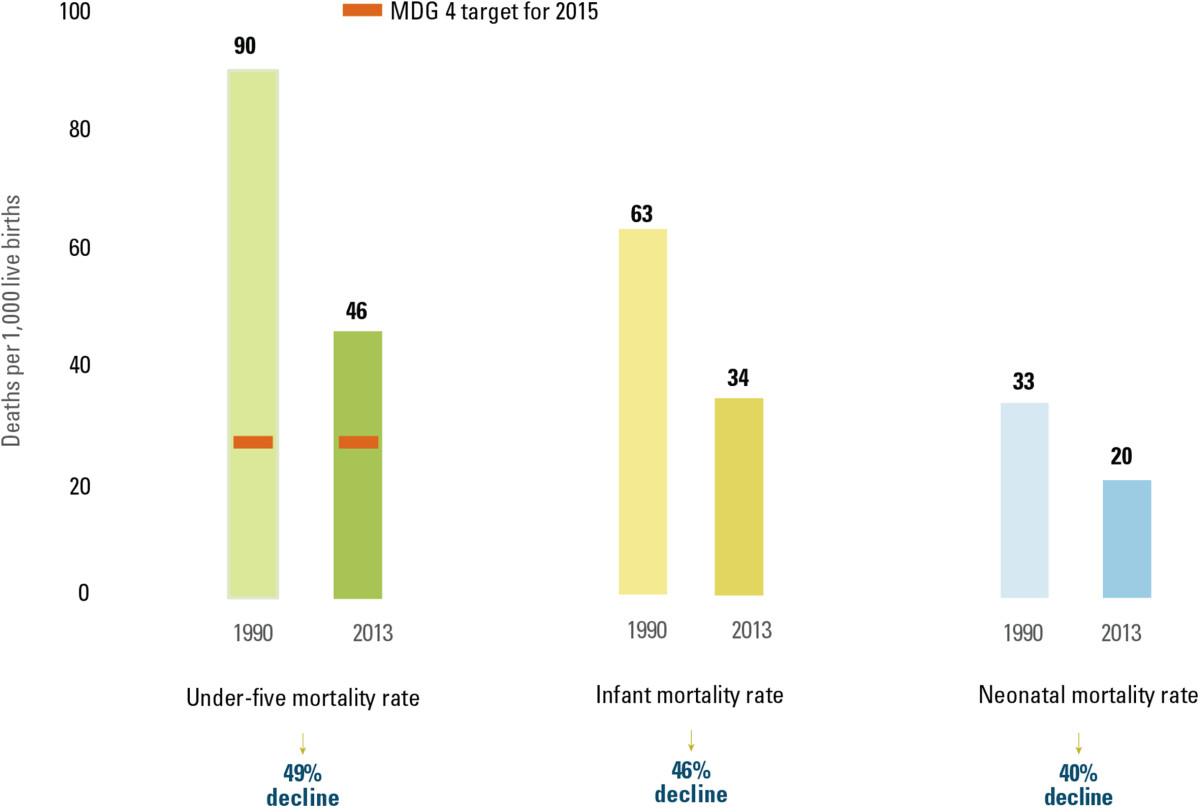
**The global under-five mortality rate fell by almost half.** Global under-five, infant and neonatal mortality rates, 1990 and 2013. Source: UNICEF, *Committing to child survival*: *A Promise Renewed progress report 2014*. New York: UNICEF; 2014.

The dramatic decline in preventable child deaths over the past quarter of a century is one of the most significant achievements in human history. In 1990, 12.7 million children under age five died. This number fell to 6.3 million in 2013, a reduction of about 50 per cent. This means that in 2013, every single day, 17,000 fewer children died than in 1990 – thanks to more effective and affordable treatments, innovative ways of delivering critical interventions to the poor and excluded, and sustained political commitment. The global progress in reducing newborn deaths is almost as striking. Between 1990 and 2013, the number of newborn babies who died within the first 28 days of life declined from 4.7 million to 2.8 million.

Despite these gains, child survival remains an urgent concern. About 17,000 children under age five died every day in 2013. Progress has been insufficient, and the target of the Millennium Development Goal 4 (MDG 4) which calls for reducing the under-five mortality rate by two-thirds between 1990 and 2015 is likely to be missed. If current trends continue in all countries, the world will not meet the target until 2026, 11 years behind schedule. At the country level, only 12 of the 60 countries with high under-five mortality rates (at least 40 deaths per 1,000 live births in 2013) are on track to achieve MDG 4 if current trends continue.

To further accelerate progress in child survival, focusing on the newborn is critical. Globally, the neonatal mortality rate fell from 33 deaths per 1,000 live birth in 1990 to 20 in 2013. However, the decline has been slower than the decline in the post-neonatal (1-59 months) mortality rate. As a result, neonatal deaths currently represent a larger share of the total under-five deaths than in 1990. In 2013, about 44 per cent of all under-five deaths occur in the first 28 days of life, increasing from 37 per cent in 1990. Every region of the world is experiencing an increase in the proportion of under-five deaths occurring in the neonatal period. In four regions, South Asia, East Asia and the Pacific, Latin America and the Caribbean, and the Middle East and North Africa – half or more of all under-five deaths are now concentrated in the first 28 days of life.

The report highlights wide disparities in global newborn survival. In West and Central Africa, for example, the risk of a baby dying within the first 28 days of life is almost 10 times higher than the risk facing a baby born in a high-income country. With 47 neonatal deaths per 1,000 live births, Angola is the riskiest place for a newborn, while Iceland and Luxembourg have only 1 neonatal death per 1,000 live births.

Encouragingly, substantial progress in some countries demonstrates that combining political commitment, sound strategies and adequate resource makes it possible to rapidly reduce neonatal mortality, regardless of national income. Although neonatal deaths are often more difficult to prevent, 80 countries have reduced the neonatal mortality rate by at least half since 1990, and 27 of those have reduced it by two-thirds or more since 1990. The largest relative gains in neonatal survival have been in nine European countries and one Asian country. The good news is that many low- and lower middle-income countries have also experienced considerable declines in the neonatal mortality rates. The 10 countries with the largest absolute declines in neonatal mortality are all low- and lower middle-income countries in Africa or Asia. The decline in these 10 countries saved the lives of 3.4 million newborns.

The report also draws attention to the urgency to focus on a healthy start to life and to provide quality care for mothers and newborns. Children who die within the first 28 days of life often do so as a result of diseases and conditions that are readily preventable or treatable with proven, cost-effective interventions. Globally, almost 3 in 4 neonatal deaths were caused by preterm birth complications (35%), complications during labour and delivery (intrapartum-related complications) (24%), and sepsis (15%) [3] (Figure 2).

**Figure 2:**
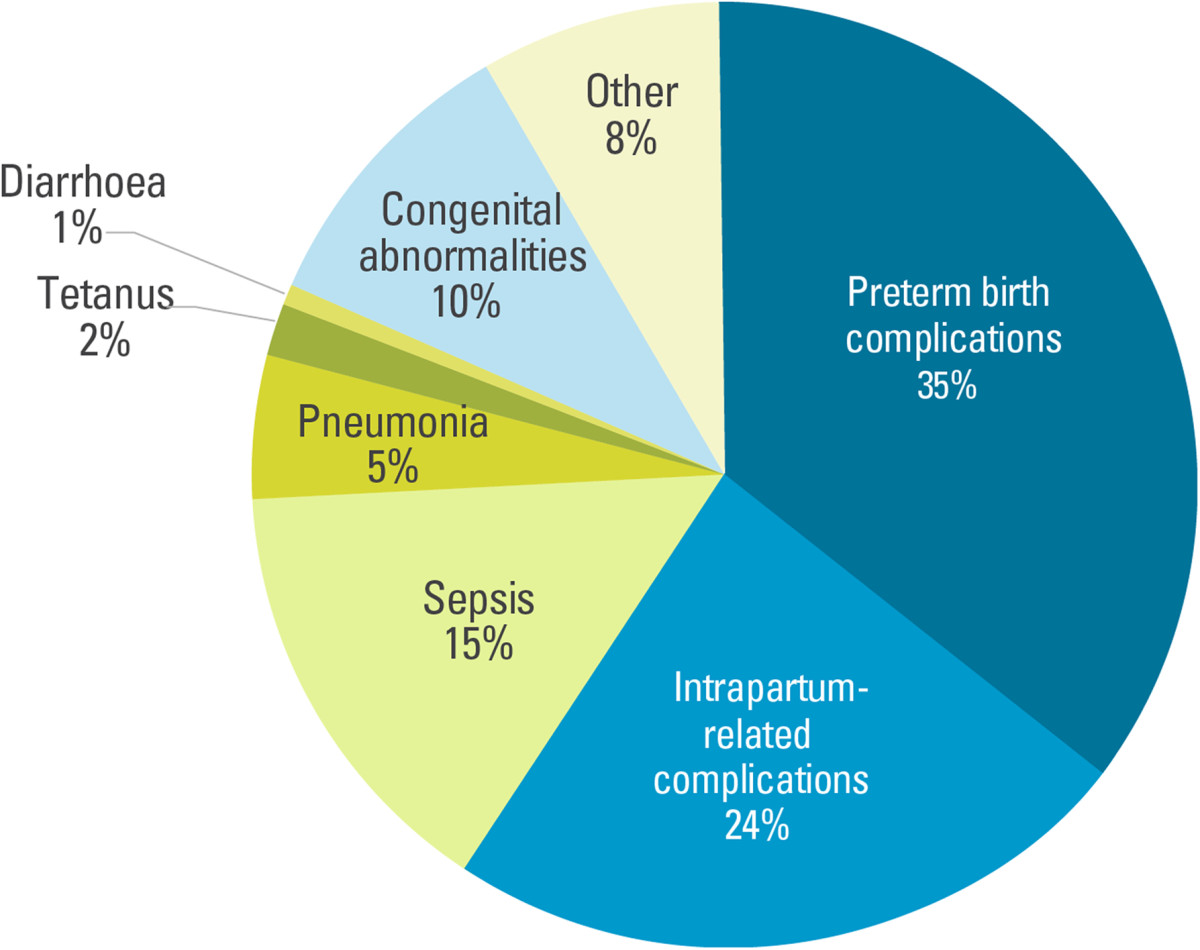
**More than a third of neonatal deaths are caused by preterm birth complications and a quarter by intrapartum-related (labour and childbirth) complications.** Global distribution of neonatal deaths, by cause, 2013. Source: UNICEF: *Committing to child survival*: *A Promise Renewed progress report 2014*. New York: UNICEF; 2014.

In addition, the first day and week are the most critical for the survival of newborns. In 2013, almost 1 million newborns died on the day they were born, accounting for 36 per cent of all neonatal deaths (Figure 3) [4]. A total of 2 million newborns died within the first seven days after birth, representing 73 per cent of all neonatal deaths.

**Figure 3:**
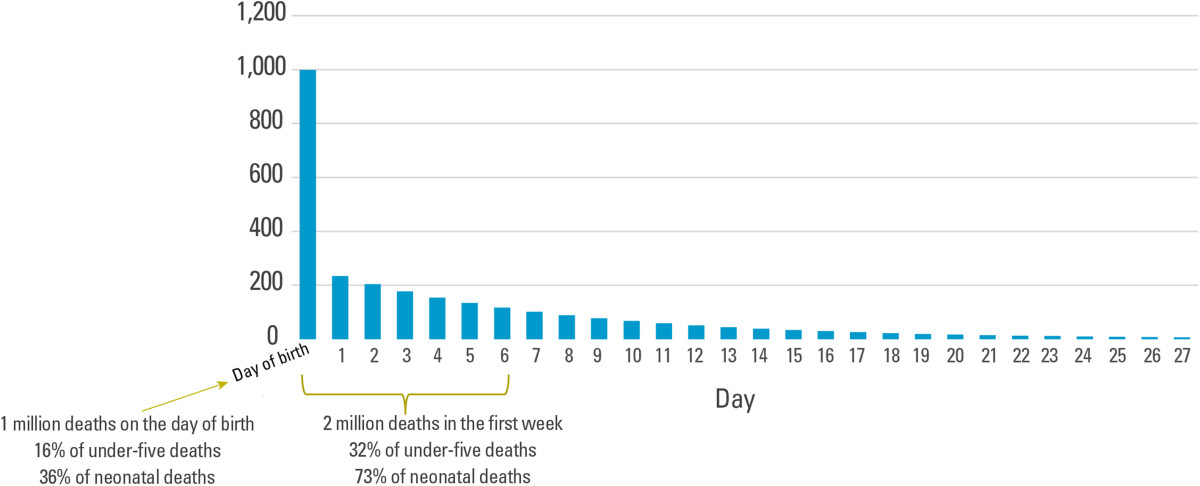
**More than a third of all neonatal deaths occur on the day of birth.** Number of deaths by day in the first 28 days of life, 2013. Source: UNICEF: *Committing to child survival*: *A Promise Renewed progress report 2014*. New York: UNICEF; 2014.

Many of these deaths are easily preventable with simple, cost-effective and high-impact interventions that address the needs of women and newborns across the continuum of care, with an emphasis on care around the time of birth. However, the UNICEF analysis using household survey data reveals that too many mothers and newborns miss out on key interventions that can save their lives [1]. Globally, only around half of all women (53%) receive the recommended minimum of four antenatal care visits during their pregnancy. In 2012, 69 per cent of births were delivered with the help of a skilled health care provider, a modest increase from a stagnant level of 57 per cent during the period 1990-2000 (Figure 4). Data on postnatal care for the newborn are lacking in many countries. In the few countries with available such data, coverage is less than 30 per cent in most. While evidence shows that initiating breastfeeding within one hour of birth reduces the baby’s risk of death by 44 per cent [5], according to recent data less than half of newborn worldwide receive the benefits of immediate breastfeeding. Less than 40 per cent of babies are exclusively breastfed throughout the first six months after birth.

**Figure 4:**
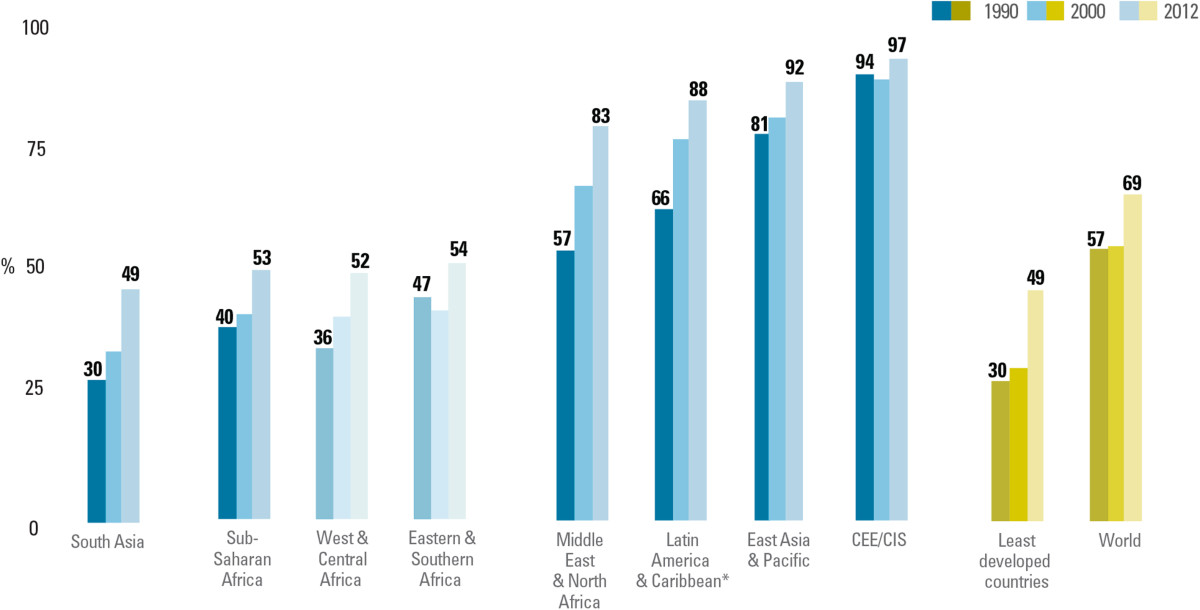
**Progress in skilled birth attendance at delivery has accelerated since 2000 across all regions, but coverage is still inadequate.** Percentage of births attended by skilled health personnel, by region, LDCs, and world, 1990, 2000, 2012. Notes: *Data for Latin America and the Caribbean refer to institutional deliveries. Estimates are based on a subset of 114 countries with available trend data for 1990–2012 covering 75% of births worldwide. Regional estimates represent data from countries covering at least 50% of regional births. Source: UNICEF: *Committing to child survival*: *A Promise Renewed progress report 2014*. New York: UNICEF; 2014.

Quality care for pregnant women and newborns is lacking, even for babies and mothers who have contact with the health system. A 10-countries analysis suggests that less than 10 per cent of mothers who saw a skilled health provider during pregnancy received a set of eight key interventions [1]. Similarly in these countries less than 10 per cent of all babies who were delivered by a skilled health professional went on to receive seven needed interventions including early initiation of breastfeeding and postnatal care.

The report calls for accelerating progress on newborn and child survival and states that more countries need to turn their pledges into practical action by sharpening national strategies for reproductive, maternal, newborn and child health, setting costed targets and monitoring progress. With millions of women and children still at risk of dying of preventable causes, maternal, newborn and child survival must remain at the heart of the post-2015 global development agenda. The world cannot abandon its promise to women and children.

## Authors’ original submitted files for images

Below are the links to the authors’ original submitted files for images. Authors’ original file for figure 1 Authors’ original file for figure 2 Authors’ original file for figure 3 Authors’ original file for figure 4
