# Supplementary figures and images for: UNICEF Report: enormous progress in child survival but greater focus on newborns urgently needed
Source: Reprod Health. 2014 Dec 6;11:82. doi: 10.1186/1742-4755-11-82 (PMC4320591; doi:10.1186/1742-4755-11-82)

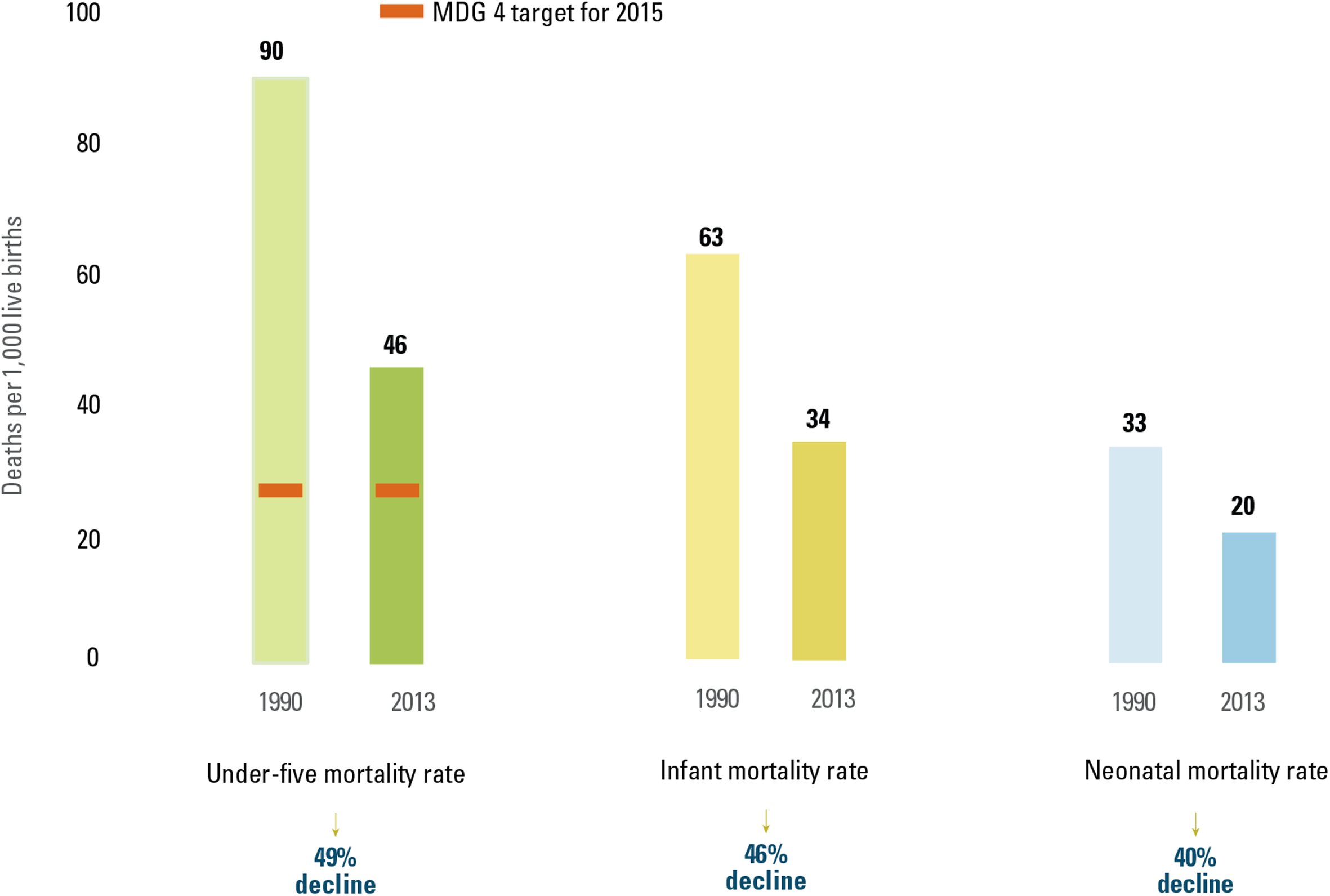

Supplement: Supplementary file 1 — Authors’ original file for figure 1 [file 12978_2014_347_MOESM1_ESM.tif]

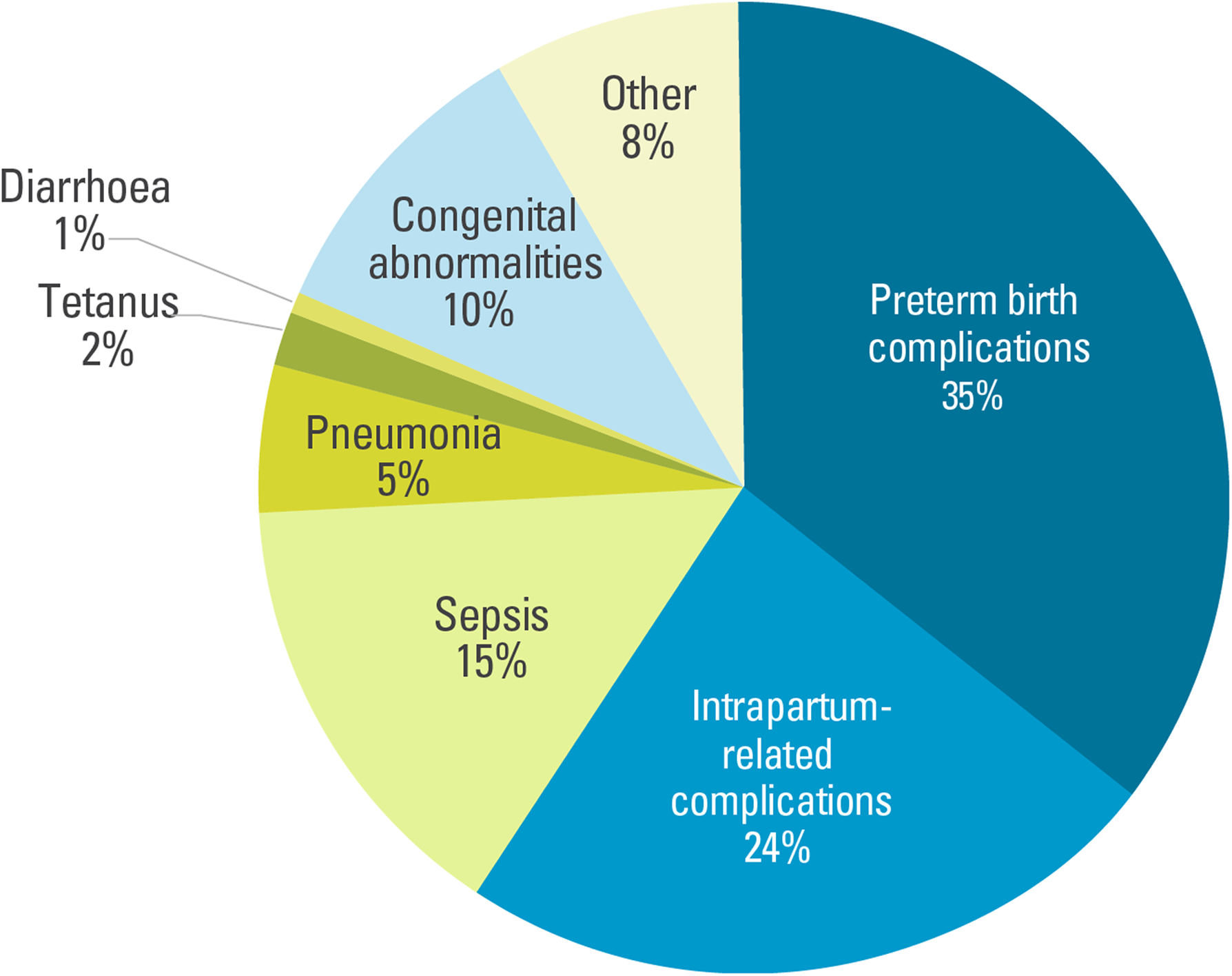

Supplement: Supplementary file 2 — Authors’ original file for figure 2 [file 12978_2014_347_MOESM2_ESM.tif]

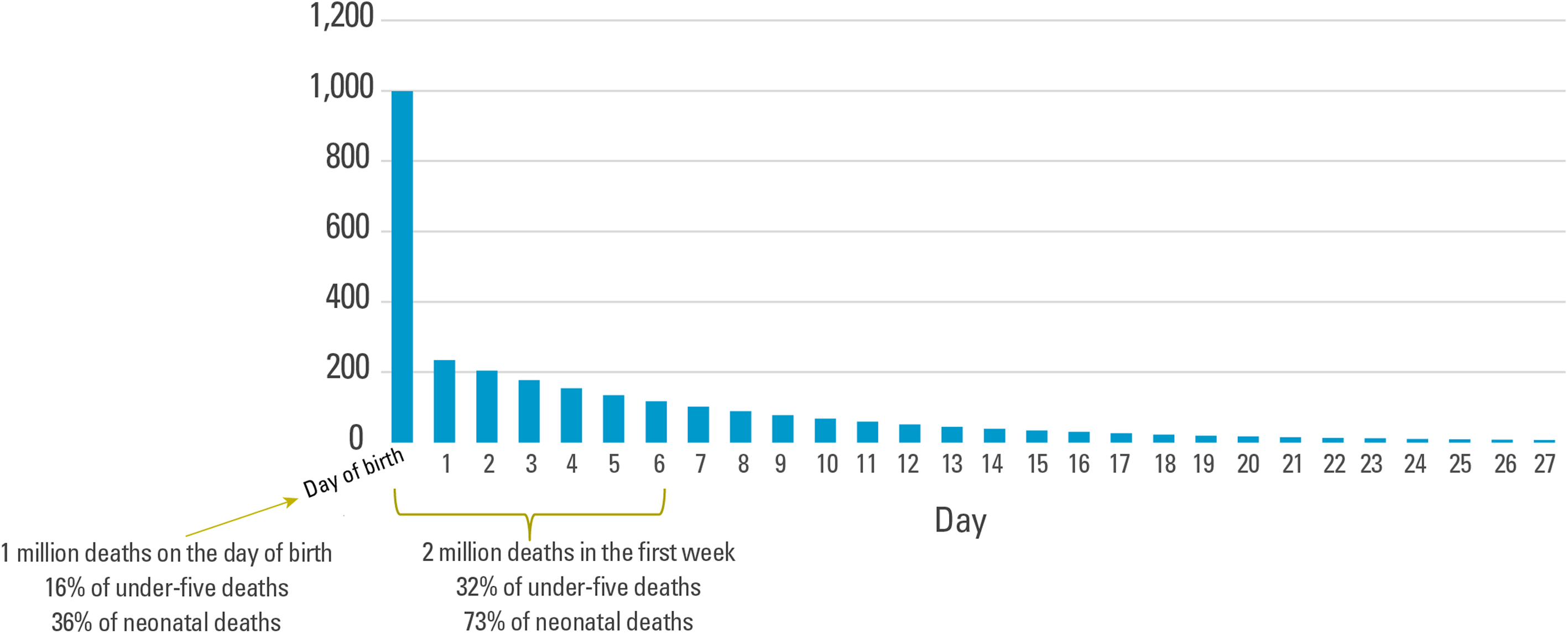

Supplement: Supplementary file 3 — Authors’ original file for figure 3 [file 12978_2014_347_MOESM3_ESM.tif]

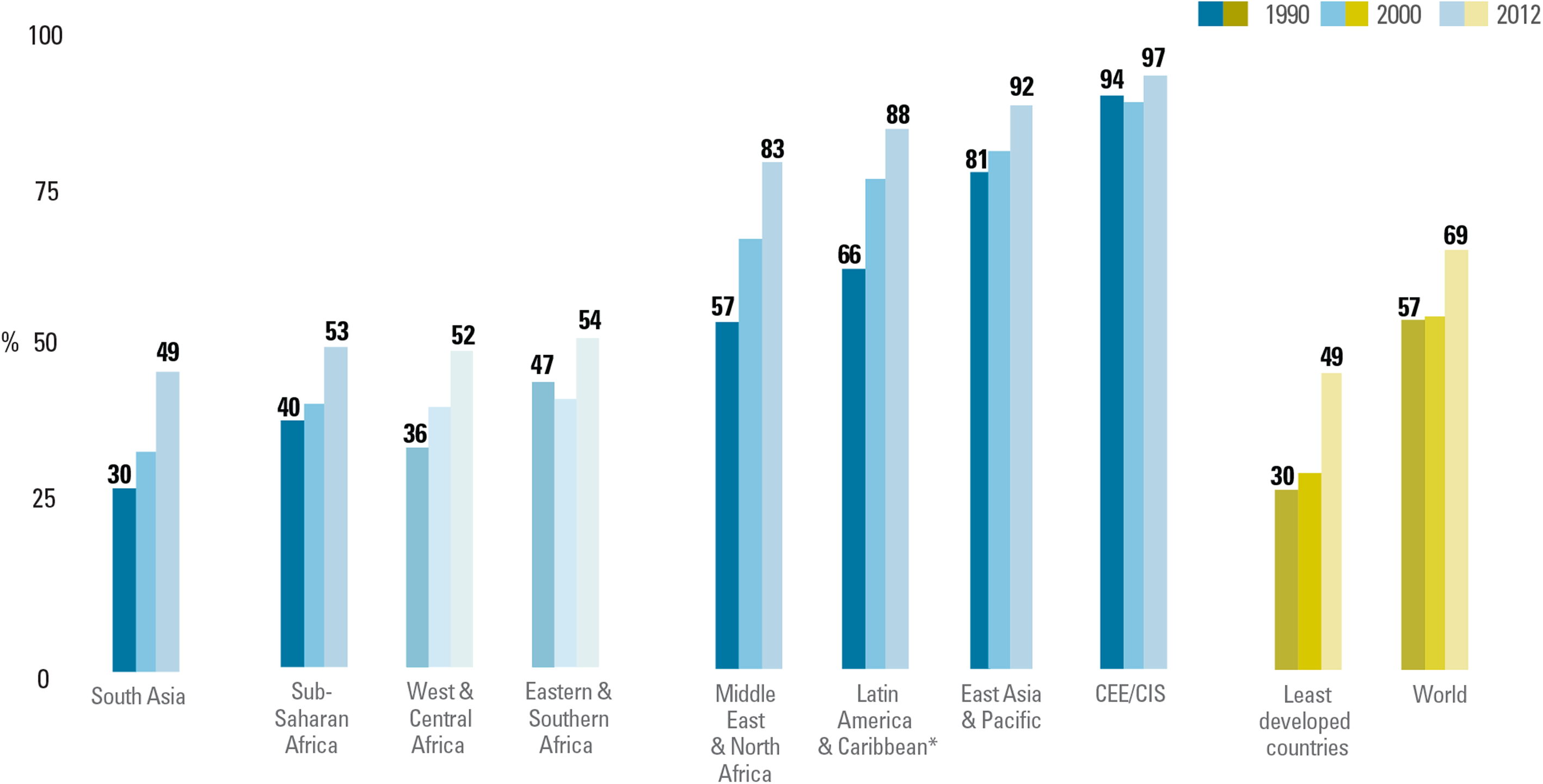

Supplement: Supplementary file 4 — Authors’ original file for figure 4 [file 12978_2014_347_MOESM4_ESM.tif]
